# 

**Published:** 1923-08

**Authors:** 


**THE DENTAL REGISTER** is published every month by the Samuel A. Crocker Company, 18 W. 7th Street, Cincinnati, O. It is mailed to subscribers during the last week in each month, being a month-end publication. Have copy for current month here by the 15th of each month. Copy received by the 15th of the month goes into that month's number of the Dental Register. Publication and business office, 18 W. 7th Street, Cincinnati, Ohio. Editorial office, 18 W. 7th Street, Cincinnati, Ohio. Address all correspondence to Editor Dental Register, Box 838, Cincinnati, Ohio, c'o The Samuel A. Crocker Company.

Notices of meetings, announcements of interest to the dental profession, and particulars of new ideas and new inventions will be given free publication in reading section. Advertising section is open to all ethical advertisements.

We are not responsible for opinions or statements made by writers whose articles this magazine may publish—nor does the publishing of any article infer in any way that we indorse the views or opinions expressed by any writer whose article we may publish.

Sal Hepatica

Materially Aids
Local Treatment
IN
PYORRHEA
Samples on Request
Bristol - Myers Co.
NEW YORK

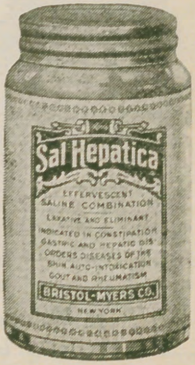

### DIRECTORY

FOR

Members of the Dental Profession whose practices are limited to a specialty.

The object of this directory is to furnish ready reference to specialists who take referred patients. If you wish your name inserted in this list please correspond with the Dental Register, Box 838, Cincinnati, Ohio.

Your name, specialty, office hours and phone number will be published.

This entire page is reserved for Professional Directory.

### Cincinnati

Dental X-Ray Diagnosis
CHARLES K. FIELD
2709 Woodburn Ave., Walnut Hills
Hours, 10 to 4
Woodburn 2486

Periodontoclasia (Pyorrhea and Oral Prophylaxis)
EDGAR H. EBERLE
605-06 Union Central Bldg., 4th and Vine Sts.
Hours, 9 to 5—Consultation hours by appointment
Main 882

Periodontoclasia (Pyorrhea and Oral Prophylaxis)
MARIE L. BECKERT
25 Groton Bldg., 7th and Race Sts.
Hours, 9 to 5—Consultation hours by appointment
Canal 842

Exodontia (Extraction and Oral Surgery)
ROBERT M. SCHELL
20 W. 9th St., between Vine and Race Sts.
Hours, 1 to 5—Consultation by appointment
Canal 118

